# Harnessing Photosynthetic ATP for Whole-Cell Biocatalysis in the Cyanobacterium *Synechocystis*

**DOI:** 10.1021/acssuschemeng.5c07236

**Published:** 2025-10-23

**Authors:** Giovanni Loprete, Eleonora Traverso, Filippo Vascon, Marco Botteri, Marina Simona Robescu, Daniela Ubiali, Laura Cendron, Tomas Morosinotto, Elisabetta Bergantino

**Affiliations:** † Department of Biology, University of Padova, Viale G. Colombo 3, I-35131 Padova, Italy; ‡ Department of Drug Sciences, 19001 University of Pavia, Viale T. Taramelli 12, I-27100 Pavia, Italy

**Keywords:** Whole-cell biocatalysis, *Synechocystis* sp. PCC 6803, Light-driven reactions, γ-Glutamyl-methylamide synthetase, *Mm*GMAS

## Abstract

Photosynthetic organisms use sunlight to produce ATP and NADPH powering their metabolism. Harnessing these products for driving biocatalytic reactions would enable development of clean and sustainable alternatives for chemical reactions. In this study, we present the demonstration that ATP produced from the photosynthetic process can fuel a biocatalytic transformation in the whole-cell configuration. This result was achieved by expressing in the cyanobacterium *Synechocystis* sp. PCC 6803 an ATP-dependent enzyme, the γ-glutamyl-methylamide synthetase from *Methylovorus mays* No. 9 (*Mm*GMAS). The expressed enzyme was able to drive, in the transgenic strain, the light-driven biosynthesis of l-theanine. Consumption of ATP by the recombinant *Mm*GMAS was even beneficial under strong illumination, protecting the photosynthetic electron transport from photodamage. These findings demonstrate the possibility of using photosynthetic microorganisms like *Synechocystis* as a potential platform for sunlight driven biotransformations with wide potential biocatalytic applications. In this perspective, we further present the tridimensional structure of *Mm*GMAS, which explains its promiscuous *in vivo* activity and provides the basis for its rational evolution.

## Graphical abstract

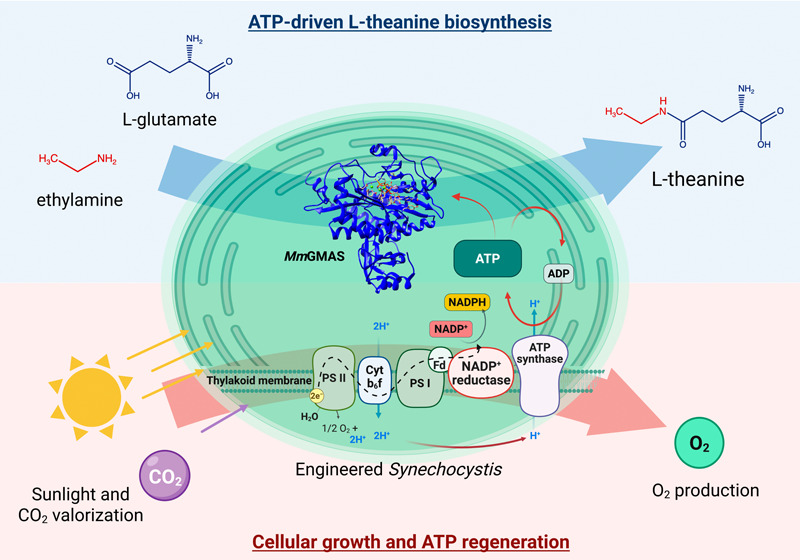

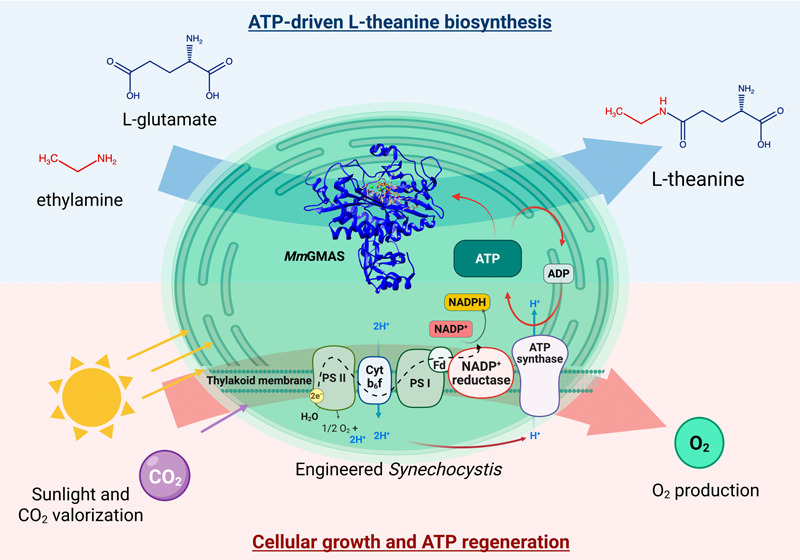

## Introduction

Cyanobacteria are photoautotrophic prokaryotes capable of converting CO_2_ into organic molecules by using light energy through photosynthesis. They are responsible for 20–30% of the earth’s organic carbon fixation and exhibit a photosynthetic efficiency that is potentially larger than that of plants.^1–3^ Cyanobacteria are a potential green chassis for biotechnological applications, bringing together advantages from the plant and bacterial worlds. While they perform photosynthesis as plants do, their metabolic networks and molecular biology share similarities with bacteria, facilitating genetic manipulations.^4^

One major possibility to be explored is the use of cyanobacteria for biocatalysis performed in the whole-cell configuration, driving chemical reactions without the need to purify enzymes, thus merging sustainability and costs benefits. A crucial aspect in the development of whole-cell biocatalytic processes is the supply of cofactors driving the desired chemical conversions. Oxygenic photosynthesis generates reducing equivalents in the form of NADPH and reduced ferredoxin (Fd) from the oxidation of water, thus representing an atom-efficient cofactor recycling system that avoids the requirement of suicide substrates and auxiliary enzymes. Consequently, researchers are currently focusing on utilizing *Synechocystis* sp. PCC6803 (*Synechocystis* from here on) to express heterologous NADPH-dependent enzymes to perform chemical reactions fueled by light through the electron transport chain.^5^ Indeed, enzymes belonging to the classes of Baeyer–Villiger monooxygenases (BVMOs), ene-reductases (ERs), imine reductases (IREDs), alkane monooxygenases, cytochrome P450 monooxygenases, and alcohol dehydrogenases have already been employed in light-fueled whole-cell biotransformations to produce high-value molecules.^6^

Besides oxidoreductases, ATP-dependent enzymes are indeed attractive biocatalysts due to their ability to drive challenging chemical reactions. Different *in vitro* ATP recycling systems have been developed such as acetate kinase–acetyl phosphate, pyruvate kinase–phosphoenolpyruvate, creatine kinase–creatine phosphate, and polyphosphate kinase–polyphosphate. The large-scale application of these systems is hindered by the high cost, the limited stability and availability of the phosphate donor, the need for multiple auxiliary enzymes for AMP-to-ATP regeneration, and the environmental impact of residual polyphosphate solutions, which contribute to plant eutrophication and substantial CO_2_ emissions.^7^

In nature, the most efficient ATP production systems are found in the processes of cellular respiration and photosynthesis.^8^ Photosystems produce ATP equivalents as well as NADPH, thus theoretically making *Synechocystis* a potential platform for the production of ATP-dependent enzymes. This possibility was explored here by expressing in *Synechocystis* a γ-glutamyl-methylamide synthetase (GMAS, EC 6.3.4.12) gene, coding an ATP-dependent enzyme that has raised interest as a biocatalyst for the production of l-theanine (l-Thea; 2-amino-4-(ethylcarbamoyl)-butyric acid). l-Thea is an unnatural amino acid contained in a wide range of nutraceutical formulations present on the market and used to increase mental focus, while providing relaxation during task performances, and to reduce stress and improve the quality of sleep.^9–14^ Currently, l-Thea has been certified as a “Generally Recognized as Safe” (GRAS) ingredient by the Food and Drug Administration (FDA), and its demand is expected to grow significantly in the next few years.

The extraction of l-Thea from its primary source, tea leaves (where it is present at about 7–21 mg/g of dry weight), remains a complex and inefficient process.^15^ Chemical synthesis methods were developed, but they are not economically competitive, while being laborious and risky, requiring numerous purification steps and the use of hazardous chemicals.^15,16^ An alternative and innovative approach for l-Thea production is its biotechnological production using enzyme-catalyzed *in vivo* biotransformations. Previous studies showed the production of l-Thea from sugar and ethylamine by fermentative processes employing a heavily engineered *E. coli* strain expressing a heterologous GMAS gene or employing two bacteria, *Pseudomonas putida* KT2440 and *Corynebacterium glutamicum*.^17–21^ The latter was able to produce up to 42 g/L of l-Thea in 48 h in a 5 L bioreactor (yield 19.6%), the highest reported yield so far.^18^

In this work, we expressed in *Synechocystis* the GMAS gene from the methylotrophic bacterium *Methylovorus mays* No. 9 (*Mm*GMAS), which codes for an enzyme that catalyzes the condensation of l-glutamic acid and ethylamine in the presence of molar equivalents of ATP as cosubstrate (Figure 1).^22^ The synthesized enzyme was functional as a biocatalyst in whole-cell configuration, driving the biotransformation of l-Thea in phototrophic conditions. Under strong illumination, the consumption of ATP by the recombinant enzyme increased the photosynthetic electron transport capacity. We finally describe the structure of the enzyme, which could elucidate its promiscuous catalytic activity in the cyanobacterial cell, opening perspective for further engineering aimed at optimizing its enzymatic activity.

**Figure 1:**
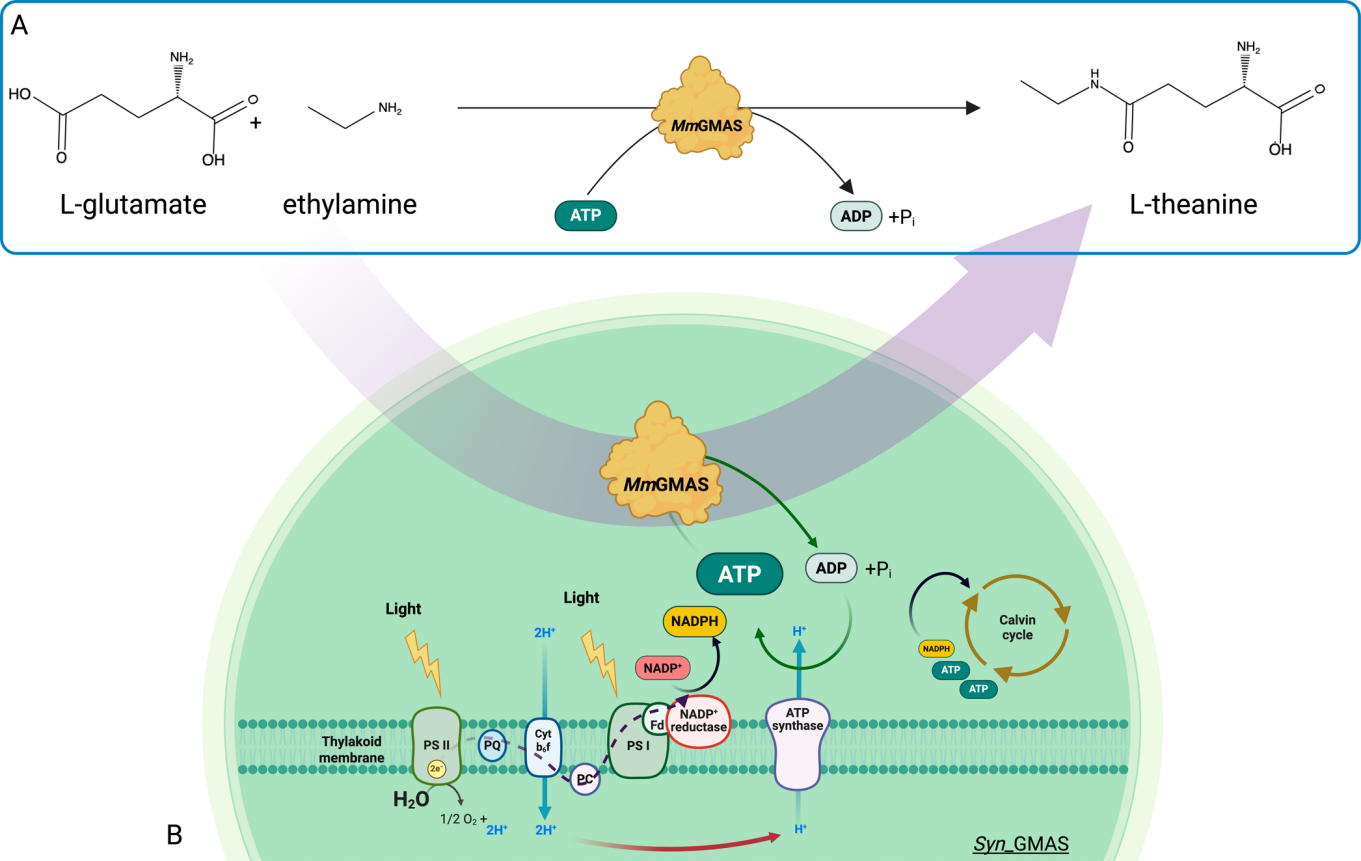
(A) *In vitro* biocatalytic reaction producing l-Thea, catalyzed by recombinant *Mm*GMAS. (B) Schematic diagram of the whole-cell biotransformation investigated in this study and performed by the *Mm*GMAS-expressing transgenic strain *Syn*_GMAS. Fluxes of electrons, NADPH and ATP cofactors, and protons (H^+^) are represented by dotted black, gray, green, and red arrows, respectively.

## Results and Discussion

### A Transgenic *Synechocystis* Strain Expressing the *Mm*GMAS Gene Produces l-Theanine from l-Glutamic Acid and Ethylamine in Whole-Cell Biotransformation

A homoplasmic, stable *Synechocystis* strain expressing the *Mm*GMAS gene under the control of the endogenous strong promoter P_cpc560_ was generated (Figure S1).^23^ Genome integration was preferred over plasmid-based gene expression since it ensures less variability and long-term application. The cyanobacterial strain, *Syn*_GMAS, was validated for the presence of the heterologous gene and protein synthesis was assessed by Western blotting (Figures S2, S3). A transgenic strain resistant to kanamycin but lacking the coding sequence for the enzyme (*Syn*_UV) was also produced and used as a control throughout the whole work (see Supporting Information).

*In vivo* biotransformations were setup to test the activity of *Mm*GMAS, which also requires (*i*) membrane permeation by the substrates provided in the growth medium and (*ii*) product secretion. Substrates, ethylamine and l-glutamic acid (l-Glu), were added to the culture medium at a final concentration of 1 mM at a working OD_730_ = 5. l-Theanine (l-Thea) formation over time was monitored by analyzing the supernatant by Thin Layer Chromatography (TLC; Figure S4A,B), which showed that l-Thea was present in the medium collected from the *Syn*_GMAS culture. Identity of the product was also confirmed by analyzing the culture supernatant by ElectroSpray Ionization-Mass Spectrometry (ESI-MS, Figure S5). These results demonstrated that the heterologous *Mm*GMAS was active and that both substrates and products can permeate the cellular membranes.

Since l-Glu is a natural amino acid, we further tested whether *Syn_*GMAS could synthesize l-Thea by providing only ethylamine, relying on endogenous l-Glu. The results of such biotransformation assays, shown in Figure S4C, confirmed that endogenous l-Glu served as the initial substrate.

### Yields and Kinetics of the Biotransformation Producing l-Theanine

Once validated, reaction kinetics were assessed using varying substrate concentrations. Aliquots of the supernatant from the biotransformations were collected at defined time points, derivatized, and analyzed by Reverse Phase-HPLC.

The samples containing 5 and 10 mM of both substrates showed comparable levels of l-Thea production and kinetic parameters (Table 1), whereas at 2.5 mM the production was reduced by approximately a half. Considering the whole cell as a biocatalyst, it is plausible that saturation of the system, either at the level of the enzyme or due to exchange equilibria, is reached at substrate concentrations around 5 mM.

**Table 1:** Measurements of Rates and Specific Activities of *Syn*_GMAS Biotransformations^c^

From data presented in Figure 2A^a^			From data presented in Figure 2B^b^		
l-glutamate and ethylamine (mM)	rate (mM/h)	specific activity (U/g_DCW_)	ethylamine (mM)	rate (mM/h)	specific activity (U/g_DCW_)
2.5	0.021 ± 0.002	0.27 ± 0.02	1	0.021 ± 0.001	0.28 ± 0.01
5	0.047 ± 0.003	0.66 ± 0.04	2.5	0.041 ± 0.001	0.57 ± 0.02
10	0.046 ± 0.002	0.64 ± 0.02			

a Data are the averages of three independent replicates. An OD_730_ = 1 corresponds to 0.24 g^–1^ mL DCW.

b Calculations were made in the interval of 0–24 h from the addition of the substrates, corresponding to the observed maximum production period.

c Calculations were made in the interval of 4–6 h from the addition of ethylamine, corresponding to the observed maximum production.

The consumption over time of substrates l-Glu and ethylamine showed that while the latter was constantly consumed from the time of its addition, the concentration of the former remained nearly constant up to 24 h suggesting that that l-Thea was initially produced by consuming endogenous l-Glu (Figure 2A). Mass balance related to l-Glu, reported in Figure S6, increased over time up to 24 h and then started to decrease, thus confirming that endogenous l-Glu was initially consumed. Mass balance related to ethylamine, showing a constant decrease, confirmed that it was steadily consumed throughout the biotransformation.

**Figure 2:**
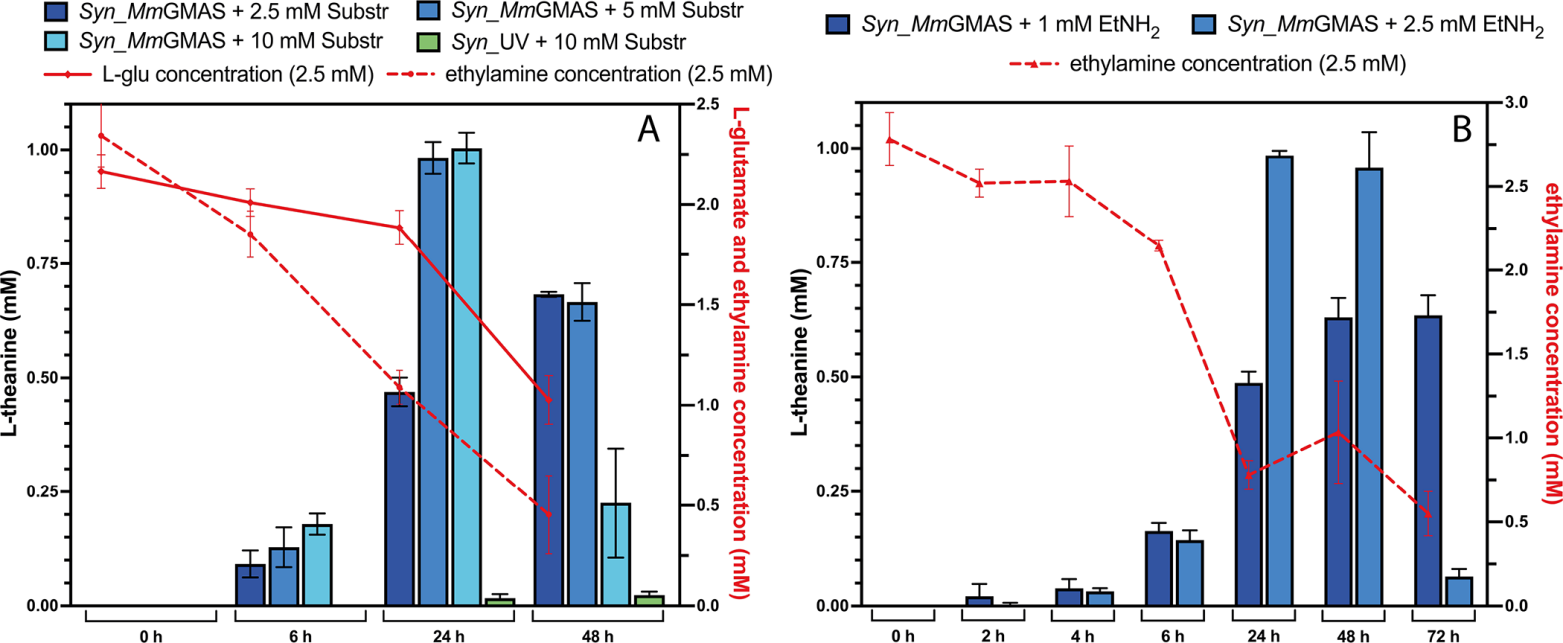
(A) Results of RP-HPLC analysis of *Syn*_GMAS whole-cell biotransformations performed at OD_730_ = 5, supplemented with different concentrations of l-glutamate and ethylamine (2.5, 5, and 10 mM) at different times. Concentration of l-Glu and ethylamine over time are represented by the continuous and dashed red lines, respectively. Control strain (*Syn*_UV) biotransformation was performed in the presence of 10 mM substrates. (B) Results of RP-HPLC analysis of *Syn*_GMAS whole-cell biotransformations performed at OD_730_ = 5, supplemented with different concentrations of ethylamine (1 and 2.5 mM) at different times. Concentration of ethylamine over time is represented by the dashed red line.

As before, biotransformations performed by adding only ethylamine confirmed the feasibility of l-Thea production relying on endogenous l-Glu (Figure 2B). Notably, starting from 2.5 mM ethylamine, l-Thea production peaked at 1 mM (174 mg/L), matching the highest yield obtained when providing both substrates in the culture medium at 5 and 10 mM. Considering that we are observing a whole-cell biocatalysis process, it is reasonable to expect variability in the presence of excess non-native molecules. Moreover, in comparison with other l-Thea production strategies employing heterotrophic hosts, our system displayed markedly lower efficiency (174 mg/L versus 42 g/L as reported by Ma et al.^18^). Nonetheless, the principal advantage of utilizing *Synechocystis* resides in its capacity to supply ATP through endogenous metabolism, albeit under conditions that remain nonoptimized.

Additionally, rates and specific activity, reported in Table 1, are similar to the ones calculated in the presence of both substrates.

The l-Thea content was also observed to decrease after 24 h from the beginning of the biotransformations at 5 and 10 mM l-Glu and ethylamine (Figure 2A). This outcome could be due either to the instability of l-Thea in the medium or to the reverse reaction catalyzed by *Mm*GMAS. Both hypotheses were excluded by *in vitro* tests (Figures S7, S8A,B). Notably, purified recombinant *Mm*GMAS was fully proficient in l-Thea synthesis, while no degradation was detectable.

A third possibility was that l-Thea could be metabolized by cyanobacterial cells. This was tested by cultivating *Syn*_GMAS and the control strain in the presence of exogenously added l-Thea. Both strains exhibited l-Thea consumption, with equivalent rates (0.016 ± 0.006 and 0.017 ± 0.013 mM/h for *Syn*_UV and *Syn*_GMAS respectively), suggesting that l-Thea is metabolized by endogenous enzymes of *Synechocystis*.

l-Thea might serve as a nitrogen source in *Synechocystis*, consistent with reports on nitrogen storage and transport in tea plants, where it is metabolized by glutaminases such as CsPDX2.1, which has been shown to be active on l-Thea.^24–27^ At least two *Synechocystis* enzymes with glutaminase activity have been reported but never tested with l-Thea.^28–30^ To test if l-Thea could be hydrolyzed and employed as an alternative nitrogen source in *Synechocystis* as well, we monitored the growth of the wild-type strain, which is unable to fix nitrogen, in a medium devoid of nitrogen, in the presence or absence of added l-Thea. Without l-Thea, wild-type *Synechocystis* rapidly showed the characteristic nitrogen-starvation phenotype (shown in Figure S9A), including the reduction of chlorophyll content (Figure S9B).^31^ Conversely, l-Thea supplementation prevented nitrogen-starvation and chlorophyll content reduction (Figure S9). This evidence confirmed that l-Thea can be metabolized and used as a nitrogen source by the cyanobacterium.

We can conclude that in biotransformations employing *Syn*_GMAS (*i*) the highest production of l-Thea (174 mg per liter of culture, 1 mM) was reached at 24 h from the addition of both substrates, l-Glu and ethylamine; (*ii*) in that time span, the production could be sustained by the endogenous reserve of l-Glu alone; (*iii*) all along the process, produced l-Thea is in equilibrium between the medium and the cells, where it is hydrolyzed by endogenous enzyme(s). For comparison purposes, the kinetics appear to be lower than those reported for other NADH/NADPH-dependent biotransformations in *Synechocystis*. For example, the lowest reported specific activity for the α-keto acid reduction of 4-methyl-2-oxovaleric acid to 2-hydroxy-4-methylpentanoic acid is 0.8 U/g_DCW_, compared to our highest value of 0.66 U/g_DCW_, though observed under nonoptimized conditions.^6^

The atom economy (AE) of the described biotransformation highlights the significant advantage of the whole-cell process in *Synechocystis*, which regenerates ATP *in situ* through photosynthesis. We calculated an AE value of 85%, which compares favorably with the 26% obtained for biotransformations that use polyphosphate kinase 2 (PPK2) and hexametaphosphate for ATP regeneration, both *in vitro* and *in vivo* (by *E. coli* cells entrapped in alginate).^32,33^

The E factor of 20185, calculated including water for both cultivation and biotransformation as described by Kourist and collaborators, reflects the relatively low level of technology readiness.^34^ It suggests that future improvement strategies should address not only the optimization of the reaction and the cellular system but also, importantly, the reduction of water consumption during cultivation in bioreactors. On the other hand, the E factor calculated excluding water is equal to 93. This metric appears more adequate to describe a biotransformation that does not produce waste, since both the cells and the spent growth broth can be recycled (e.g., the former as fertilizers, the latter as base solution for subsequent cultivations) (see Supplementary Table S1 for calculation details).^35^

### *Mm*GMAS Affects the Cyanobacterial Metabolism, Leading to a Decreased Growth Rate

Once the feasibility of the biotransformation was verified, we investigated the impact of the heterologous protein on the *Synechocystis* growth. Figure 3A shows that, in the absence of added substrates, *Syn*_GMAS and control *Syn*_UV cultures showed similar growth and biomass accumulation (1.23 ± 0.16 g_DCW_/L vs 1.30 ± 0.15 g_DCW_/L respectively). However, *Syn*_GMAS showed a small delay in growth. This could be ascribed either to the metabolic burden due to *Mm*GMAS (over)­production or to its basal activity on one or more endogenous molecules, thus consuming ATP. The enzyme has in fact been reported to be a promiscuous catalyst, capable of producing many different γ-glutamyl compounds from ammonia up to more sterically hindered amines, such as tryptamine or phenylethylamine.^22,36^ Considering the presence of various endogenous amines in *Synechocystis*, e.g. ammonia, (see Figure S10), a basal activity by the heterologous *Mm*GMAS is likely.

**Figure 3:**
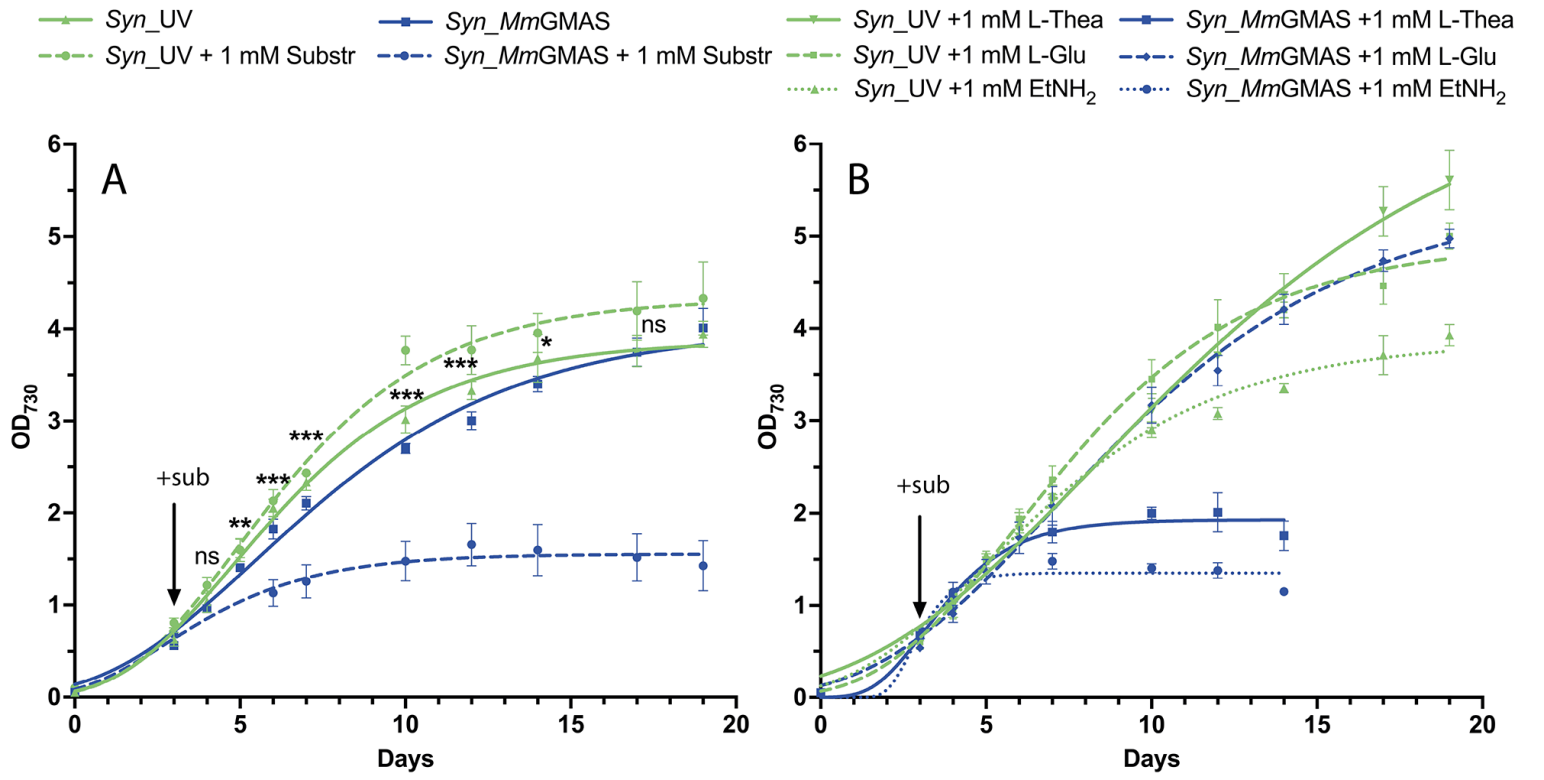
Effects of substrate addition in recombinant *Synechocystis* growth. (A) Curves comparing growth rates of *Syn*_GMAS and *Syn*_UV strains in the absence and presence of 1 mM l-glutamate and ethylamine. Statistical significance (two-sample *t* test) is represented by ns (not significant) and asterisks (significant, *p < 0.05, **p < 0.01, ***p < 0.001) and refers to each time-point measured in the absence of substrates. (B) Curves comparing growth of strain *Syn*_GMAS and *Syn*_UV in the presence of 1 mM l-glutamate, l-theanine, and ethylamine separately. Cultures (three independent biological replicates for each strain and each condition) were cultivated in flasks under continuous shaking and under standard cultivation conditions.

When substrates were added to the medium, however, the growth profile of the two strains significantly changed, and while the presence of both l-Glu and ethylamine are well tolerated by the control, their addition drastically inhibited *Syn*_GMAS growth (Figure 3A).

Individual substrates or products were added separately to both *Syn*_UV and *Syn*_GMAS (Figure 3B). Ethylamine did not impact *Syn*_UV growth, while l-Glu and l-Thea had an evident beneficial effect (Figure 3B). In contrast, *Syn*_GMAS exhibited a positive response exclusively to the addition of l-Glu, while its viability was compromised by the presence of either ethylamine or l-Thea. This suggests that growth inhibition is associated with the activity of the heterologous enzyme in the presence of ethylamine, whether it is externally supplied or generated via l-Thea hydrolysis.

### *Syn*_GMAS Is Characterized by Decreased Endogenous Free ATP Content and Enhanced Photosynthetic Electron Transport Rate

In cyanobacteria, the light-excited electrons drive the reduction of NADP^+^ into NADPH and contribute to the establishment of a proton gradient, then exploited by the ATP synthase to produce ATP, sustaining cellular growth. We thus monitored the impact of the ATP consuming enzyme on endogenous ATP levels and photosynthetic efficiency.

Intracellular ATP levels are 10% lower in *Syn*_GMAS compared to *Syn*_UV (Figure 4A) which could explain the delay in growth rate observed for *Syn*_GMAS. In biotransformations (performed at 150 μmol photons·m^–2^·s^–1^), 2 h after substrate addition, ATP consumption is strongly enhanced (Figure 4B), further confirming enzyme activity and explaining the growth inhibition of *Syn*_GMAS in the presence of ethylamine (with or without l-Glu, Figure 3A,B). These results clearly confirm that the enzyme activity is ATP-dependent.

**Figure 4:**
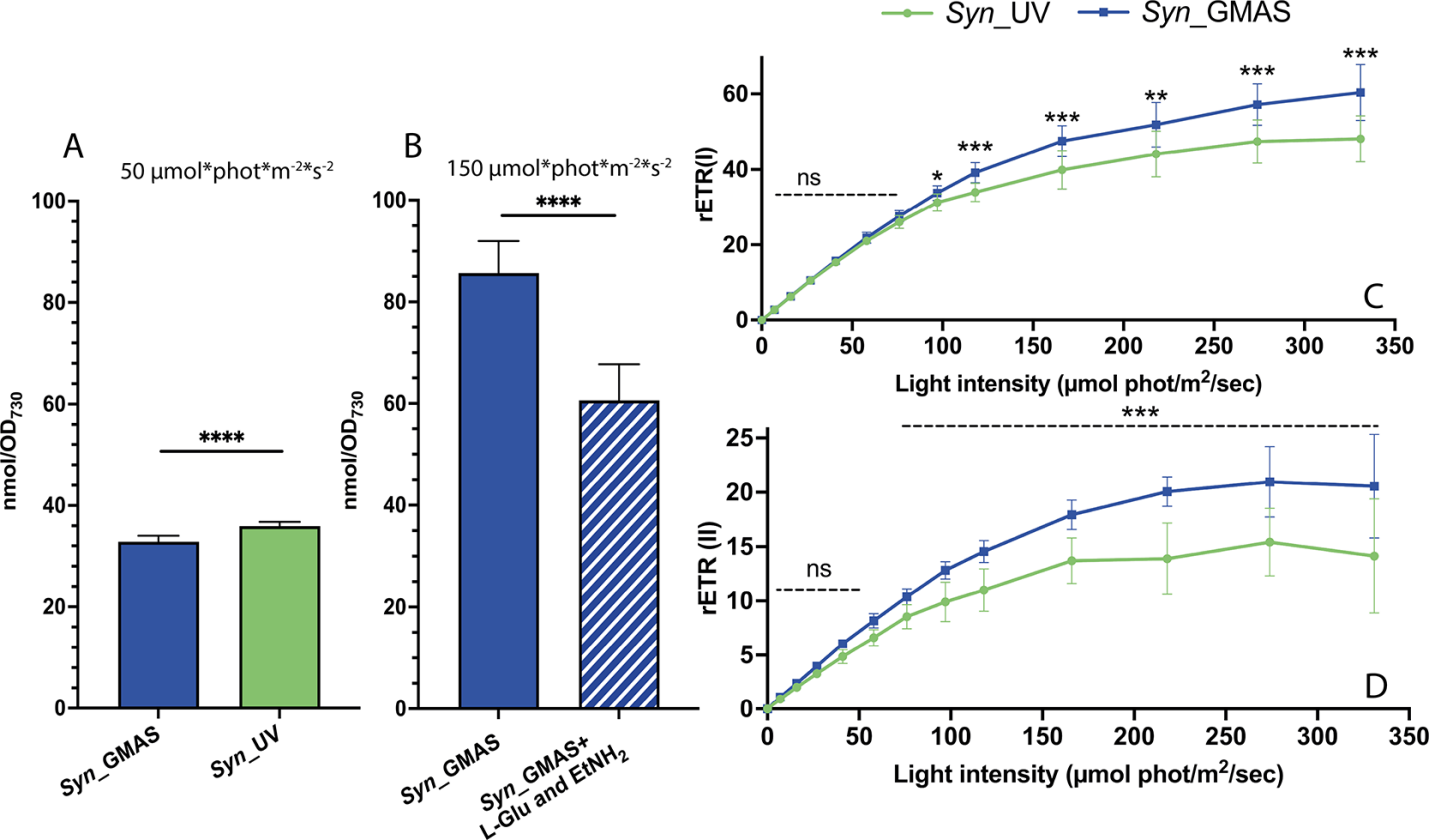
ATP content and electron transport rates of PSII and PSI (rETR). (A) ATP content of *Syn*_GMAS and *Syn*_UV grown in standard conditions (up to exponential phase, in BG11 and under 50 μmol photons·m^–2^·s^–1^) in the absence of exogenous substrates. (B) ATP quantification of *Syn*_GMAS whole-cell biotransformations after 2 h of cultivation in the presence or absence of 1 mM substrates, in BG11 and under 150 μmol photons·m^–2^·s^–1^ constant illumination. (C, D) Measurements of relative electron transport rate of PSI and PSII, (C) rETR­(I) and (D) rETR­(II), respectively, in *Syn*_GMAS and control *Syn*_UV strains. Statistical significance (two-sample *t* test) is represented by ns (not significant) and asterisks (significant, *p < 0.05, **p < 0.01, ***p < 0.001 and **** *p* < 0.0001).

Maximal photosynthetic efficiency (*F*_v_/*F*_m_) was evaluated in dark adapted *Syn*_UV and *Syn*_GMAS strains grown in the absence of any substrate and showed no significant differences (0.30 ± 0.04 and 0.31 ± 0.06 respectively). When exposed to increasing light intensity, the relative PSII electron transport rate (rETR­(II)) was higher for *Syn*_GMAS than for the control (Figure 4D).

Similarly, the PSI-associated electron transport capacity (rETR­(I)) was higher in *Syn*_GMAS under higher light intensities (Figure 4C). This increase can be ascribed solely to the presence of the heterologous enzyme consuming ATP. Through this activity, ATP consumption is enhanced, stimulating a higher electron transport rate. As a result, the saturation of photosynthetic ETR at high light intensities is reduced, which can be beneficial to protect from photodamage.

### Structural Characterization of *Mm*GMAS

The data presented above suggest a promiscuous behavior of *Mm*GMAS *in vivo*. To gain deeper insights into its catalytic activity and potential for optimization in biocatalytic applications, the determination of its tridimensional structure was thus pursued. Currently, the only experimental structure belonging to this enzyme class is the one of the more specific GMAS from *Rhodovulum* sp. 12E13 (*Rh*GMAS; PDB: 7CQL, 7CQN, 7CQQ, 7CQU, 7CQW and 7CQX).^37^ *Rh*GMAS is indeed a more efficient enzyme on its preferred substrate methylamine (also a substrate for *Mm*GMAS) and on ethylamine but exhibits a more restricted substrate scope compared to *Mm*GMAS.^36,37^

*Mm*GMAS was recombinantly produced and purified from *E. coli* cultures (Figures S11A,B, S12); we obtained the crystal structure of the complex *Mm*GMAS–ATPγS at 2.65 Å (PDB 9QUR; Table S4), showing that the enzyme is organized as a homo-dodecamer composed of 2 stacked hexameric rings of *Mm*GMAS subunits (Figure 5A and 5B). Details of interactions among protein chains are illustrated in the Supporting Information (Results section and Figure S13).

**Figure 5:**
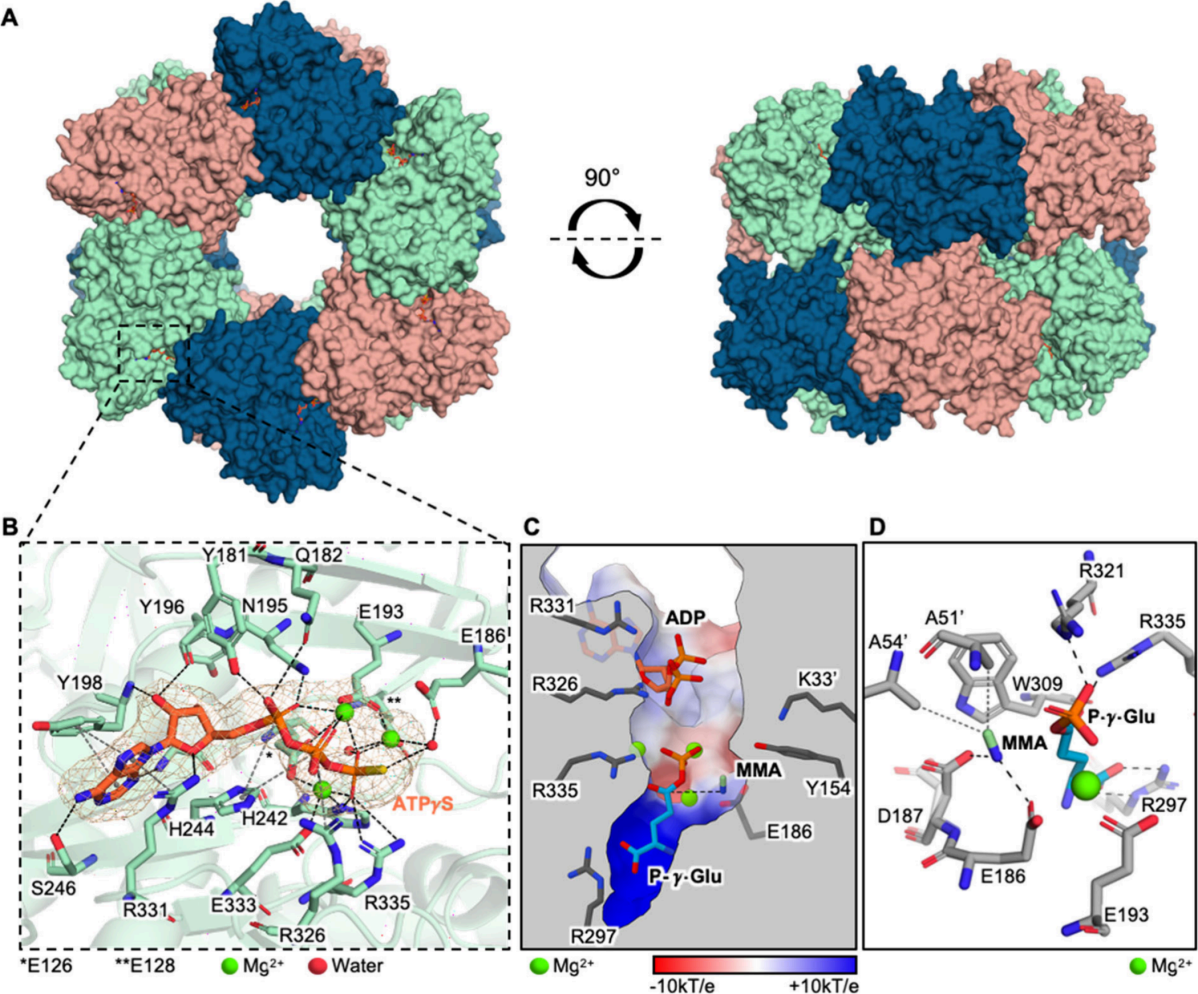
Crystal structure and computational modeling of *Mm*GMAS. (A) Front and top view of the *Mm*GMAS homo-dodecamer (PDB 9QUR). (B) Interactions between ATPγS, Mg^2+^, and *Mm*GMAS residues. Omit map of ATPγS and Mg^2+^ cations at 2.5σ is displayed. H-bonds and metal coordination bonds are displayed as black dashed lines; salt bridges, π-stacking, and cation−π interactions are shown as gray dashed lines; hydrophobic interactions are depicted as gray dotted lines. (C) In silico generated model of the quaternary complex *Mm*GMAS/ADP/P-γ-Glu/MMA. Color gradient depicts the surface electrostatic potential of the *Mm*GMAS active site. The dashed line represents the nucleophilic attack of the MMA amine group on P-γ-Glu Cδ. (D) Interactions between *Mm*GMAS residues, MMA, and P-γ-Glu. Hydrophobic interactions are depicted as gray dotted lines, while electrostatic contacts are shown as black dashed lines.

*Mm*GMAS shares 44% sequence identity (59% similarity) with the primary sequence of *Rh*GMAS; high conservation of the tridimensional fold and residues of the active site is also observed. The most notable structural differences are found in flexible secondary structure elements which, despite not being directly involved in the catalytic cleft of *Mm*GMAS, are located both upstream and downstream of the substrate and cofactor binding sites (the β-hairpin 132–143 and the random-coil loops 248–252 and 256–267). These regions might impact catalytic activity, protein stability, and dynamics through epistatic effects. These observations support the hypothesis that the substrate specificity of wild-type *Mm*GMAS relies on the plasticity of the catalytic site and the hexameric assembly, likely explaining its reported higher promiscuity compared to *Rh*GMAS.^36,37^

To better understand substrate binding in *Mm*GMAS, a quaternary complex model including ADP, monomethylamine (MMA), and l-γ-glutamyl phosphate (P-γ-Glu) was generated. Compared to the previously proposed model, the one presented in this study shows MMA occupying a novel binding site closer to the Cδ of P-γ-Glu, positioning it effectively for nucleophilic attack and thereby facilitating amine addition (Figure 5B and Supporting Information). This binding mode is stabilized by hydrophobic and electrostatic interactions involving key residues Ala51′ and Ala54′ (Figure 5D). Moreover, molecular dynamics simulations confirmed greater stability of MMA in this newly identified site compared to the previous model (Supporting Information and Figure S14). These findings are supported by prior mutagenesis studies, particularly highlighting the essential role of Glu186 and Asp187 (Glu179 and Asp180 in *Rh*GMAS; Figure 5D), underscoring the functional relevance of the proposed binding mode for *Mm*GMAS.^37^ Besides explaining the observed promiscuity in *Synechocystis*, the experimentally determined X-ray structure of *Mm*GMAS, together with these modeling and dynamics studies, furnishes valuable insights for further engineering and evolution of this biocatalyst for both *in vivo* and *in vitro* applications.^32^

## Conclusions

This study demonstrates the feasibility of a whole-cell biotransformation approach that exclusively relies on photosynthetically generated ATP. Our *Synechocystis* strain expressing the *Mm*GMAS gene, is capable of producing l-Thea when cultivated in minimal medium and supplemented with the required substrates.

As is commonly observed with recombinant enzymes produced in active form within host cells, *Mm*GMAS was found to affect cellular metabolism by consuming endogenous ATP, thereby altering metabolic equilibria and leading to reduced growth rates. Notably, despite this metabolic perturbation, the cyanobacterium was still able to sustain the ATP-dependent activity of *Mm*GMAS producing l-Thea for up to 24–48 h. By harnessing photosynthesis, the need for sacrificial cosubstrates is eliminated, thereby improving atom economy, as observed for other biotransformations carried out in *Synechocystis*.^34^ From the perspective of developing a sustainable, scalable process, aimed at reducing the E factor, high-throughput optimization will be required to minimize water and energy consumption, while maximizing reaction yields and improving both cultivation systems and product purification.

The selected enzyme and the specific catalyzed reaction were instrumental in establishing ATP as a viable energy currency for driving biocatalysis in *Synechocystis*. ATP is a central metabolite for the cell involved in most metabolic pathways. Photosynthetic organisms use multiple strategies to adjust its availability and NADPH/ATP ratio. In cyanobacteria, both cyclic and pseudocyclic electron flows contribute, while respiration also consumes reducing power for the production of ATP. In this work we demonstrate that cells are able to provide ATP for the bioconversion, thus affecting bioenergetic pathways that will be investigated.

This demonstration represents a breakthrough in the valorization of this organism as a promising platform to exploit sunlight and CO_2_ to sustainably produce high value chemicals. Despite having been extensively studied as a model for the structures and functioning of the photosynthetic apparatus, cyanobacteria are currently not an industrially relevant chassis: their molecular biology tools are far from being well developed and easily applicable for biotechnological applications. Fundamental research and genetic manipulation, focused on enlarging molecular toolkits, enhancing light-harvesting capabilities, optimizing the operation of photosystems (PSs), minimizing parallel side reactions, and enabling structure-driven rational evolution of the enzyme(s), are still required to address factors that limit the transition to industrially relevant applications, in a profitable, fair competition with well-established heterotrophic systems.^38^

## Methods

### Enzymes and Reagents

Polymerases, restriction enzymes, DNA oligonucleotides (Table S2), standard l-theanine, and l-glutamate were purchased from Thermo Fisher Scientific (United States). The *Mm*GMAS coding sequence was purchased from GeneArt, Thermo Fisher Scientific (United States). Standard ethylamine and other reagents were purchased from Merck (Merck Life Sciences, Germany).

### Bacterial Strains, Cloning, and *Synechocystis* Transformation

The following strains were used: *Synechocystis* sp. PCC 6803 purchased from the Pasteur Culture collection of Cyanobacteria (PCC, France); *E. coli* DH-5a and BL21 (DE3) purchased from New England Biolabs (United States). Cloning in *E. coli* and PCR amplifications were performed by routine methodologies. Plasmid pSuperP_*Mm*GMAS (Figure S1) was constructed starting from the *Synechocystis* empty plasmid pSuperP_UV.^23^ *Nco*I and *Not*I restriction sites at the 5′ and 3′ termini, respectively, were used to clone *Mm*GMAS coding sequence fused to an N-terminal 6XHisTag. The protocol for *Synechocystis* transformation, using plasmid DNA digested by *Dra*I, was based on phosphate deprivation.^39^

### *Synechocystis* Standard Cultivation Conditions, Optical Density Measurements, and Dry Cell Weight

Standard culture cultivation was performed in BG11 medium (Table S3) with continuous shaking (150 rpm) and under constant light illumination, 50 μmol photons·m^–2^·s^–1^. Cultivation flasks and Corex tubes used for biotransformations were covered with a hydrophobic cotton cap, allowing air exchange while limiting medium evaporation. Cyanobacterial population density was estimated from the turbidity of the culture, typically expressed as Optical Density at 730 nm (OD_730_). Dry cell weight (DCW) was measured by completely drying 10 mL of cyanobacterial cultures on 0.2 μM nylon filters, which were dried overnight at 60 °C.

### Total Protein Extraction from *Synechocystis* Cells and Western Blot Analysis

Expression of the *Mm*GMAS gene in *Syn*_GMAS was verified by Western blotting.^23^ Briefly, total protein extract was obtained by harvesting cell cultures in exponential growth phase by centrifugation, washing and resuspending the pellet in resuspension buffer (50 mM HEPES–NaOH, pH 7.5, 30 mM CaCl_2_, 800 mM sorbitol, 1 mM ε-amino-n-caproic acid) and then homogenizing using One Shot Cell disruptors (Costant Systems, United Kingdom). After centrifugation, soluble protein content was quantified by Bradford assay (SERVA Electrophoresis GmbH, Germany) and 10 μg was run in 12% UREA-PAGE. Western Blot was performed using primary mouse monoclonal anti-His-Tag antibody, HRP-conjugated (SB194b, Southern Blotting, USA). A VWR Imager CHEMI Premium was used for chemiluminescence detection (VWR International s.r.l., Italy).

### Chlorophyll Extraction and Quantification

*Synechocystis* wild-type was cultivated in modified BG11 (nitrogen depleted medium, Table S3) in the presence and absence of l-theanine. After 48 h, cultures were diluted by a factor of 10 to estimate the cellular content. Samples (20 μL) were deposited in the cell counting chamber, allowed to settle, and counted using the Cellometer Auto X4 Cell Counter (Nexcelom Bioscience, United States). Counts were performed three times on three independent replicates. In parallel 2 mL of each replicate was harvested by centrifugation for 20 min at 6000*g*. After removal of the supernatant, cells were resuspended in *N*,*N*-dimethylformamide (DMF) and left overnight at 4 °C in darkness to extract chlorophylls. Then, the solutions were harvested by centrifugation for 15 min at 6000*g* to separate cellular debris and quantified using a Cary60 UV–vis spectrophotometer (Agilent, United States).

### Spectroscopic Analyses

Fluorescence and P700 measurements were carried out with a Dual-PAM-100 fluorometer (Walz-Germany) on samples grown up to early exponential phase (OD_730_ = 1.5–2) under constant light illumination of 60 μmol photons·m^–2^·s^–1^. Prior to the analyses, samples were dark-incubated for 4 min and measurements were performed on a cell suspension of OD_730_ = 2 with sample loaded in the 1 cm rectangular quartz cuvette.^40^ Samples were exposed to increasing intensities of actinic red light and PSII- and PSI-related parameters were calculated upon saturating pulses of 5000 μmol photons·m^–2^·s^–1^, 600 ms.^40,41^ *F*_v_/*F*_m_ = (*F*_m_ – *F*_0_)/*F*_m_ was determined as the maximum quantum efficiency of PSII in the dark-adapted state. The relative electron transport rate of PSII and PSI (respectively denoted as rETR­(II) and rETR­(I)) were estimated from PSII and PSI yield (*Y*(II) and *Y*(I)) calculated respectively as *F*_v_′/*F*_m_′ = (*F*_m_′ – *F*_0_)/*F*_m_′ and 1 – *Y*(ND) – *Y*(NA) (where *Y*(ND) = *P* – *P*_0_/*P*_m_ and *Y*(NA) = *P*_m_ – *P*_m_′/*P*_m_.^42^

### Intracellular Free ATP Quantification

Intracellular free ATP was measured by the ATP Determination kit (A22066, Thermo Fisher Scientific, United States). *Syn*_GMAS and *Syn*_UV strains were grown with continuous shaking at 150 rpm and under standard cultivation conditions up to early exponential growth phase (OD_730_ = 1.5–2). Aliquots of samples (150 μL) were immediately frozen in liquid nitrogen. After the addition of an equal volume of glass beads (150–212 μm, Sigma G-1145), homogenization was performed using the Bullet Blender Storm Pro cell homogenizer (Next Advance, United States). Samples were then centrifuged 1 min at 20000*g* at 4 °C. ATP quantification was performed according to the manufacturer’s instructions.

### TLC and ESI-MS

Thin-Layer Chromatography (TLC) silica glass plates 60G F_254_ (Merck Life Sciences, Germany) were used. The mobile phase was set on the basis of literature and experimental trials. Finally, the eluent NH_3_/EtOH (70/30) for l-glutamate, ethylamine, and l-theanine separation was used. Detection was performed using 0.1% w/v ninhydrin in EtOH under heat.

For mass analysis, supernatant samples collected (2 mL) were dried under N_2_ flow and then suspended in MeOH (300 μL). Subsequently, the mixture was centrifuged to eliminate insoluble residues and then diluted 1:10 in MeOH for direct MS injection and analysis. MS detection was performed by using a linear ion trap mass spectrometer (LTQ) equipped with an electrospray ion source (ESI) (Thermo Scientific, San Jose, CA, USA) and controlled by X-calibur software (2.0.7 version). The following MS parameters were applied: positive ion mode, scan range 150–1000 *m*/*z* in full scan mode, source voltage 4.6 kV, capillary voltage 30 V, sheath gas flow rate 10 (arbitrary units), auxiliary gas flow rate 4 (arbitrary units), capillary temperature 250 °C, and tube lens voltage 80 V.

### Supernatant Derivatization and RP-HPLC Analysis Protocol

Supernatant samples for RP-HPLC were isolated after centrifugation of culture samples at 13000*g* for 15 min. 120 μL of methanol was added to 60 μL of supernatant. After 25 min centrifugation at 13000*g*, samples were derivatized before RP-HPLC analysis. The protocol was optimized starting from the one described in Perucho et al., 2015.^43^ Derivatization was performed by mixing 150 μL of samples with 75 μL of derivatization mix (32 mg of *o*-phthaldialdehyde dissolved in 800 μL of methanol, 7140 μL of 0.4 M borate buffer, pH 9.5, and 60 μL of 3-mercaptopropionic acid). After 5 min at room temperature, the derivatization was quenched by the addition of 37.5 μL of 5% v/v acetic acid and then immediately analyzed. RP-HPLC analyses were carried out on a Nucleosil 100 C-18 column (25 cm × 4.6 cm, 5 μm) (Altmann Analytik, Germany), employing as mobile phase A 5% methanol/95% sodium acetate, 0.05 M pH 5.88, and mobile phase B 70% methanol in water. After injection (20 μL) in the Beckman Gold HPLC system (Beckman Coulter, United States), the analysis was performed by a gradient elution as follows: 0–13 min (A 75%–B 25%→ A 0%–B 100%), 14–15 min (A 0%–B 100%), 16–20 min (A 0%–B 100% → A 75%–B 25%), 21–25 min (A 75%–B 25%).

### Atom Economy and E Factor Calculations

Atom economy was calculated according to Constable et al., 2002,^44^ including either water or hexametaphosphate molecular weight as reagents for reactions performed in *Synechocystis* or in vitro/*E. coli* cells, respectively.^32,33^ MW of water was approximated to 12, considering a stoichiometry of 3 ATP molecules produced for 2 H_2_O molecules consumed in the photophosphorylation process. The E factor was calculated by accounting for water consumption in both cultivation and biotransformation steps (see Supporting Information), following the approach described Grimm et al., 2025.^34^

### Protein Overproduction and Purification

The coding sequence of *Mm*GMAS with an N-terminal 6xHis-Tag was cloned in the pET28a­(+) plasmid by *Nco*I and *Not*I restriction digestion and following ligation. Recombinant *Mm*GMAS was produced in *E. coli* BL21 (DE3) (New England Biolab, United States) transformed with pET28_*Mm*GMAS. Preparative cultures were carried out in 1 L of LB medium, and the cells were grown in a shaking incubator (180 rpm) at 37 °C to an optical density at 600 nm (OD_600_) of 0.4–0.6. Protein synthesis was induced by adding 0.2 mM isopropyl-β-d-1-thiogalactopyranoside (IPTG) and prolonged overnight at room temperature. Cells were harvested by centrifugation (4 °C, 15 min, 5000*g*) and resuspended in 50 mM sodium phosphate, pH 7.0, 10% v/v glycerol. Cell lysis was accomplished using a French Press (One Shot Cell Disruptor; Constant Systems, United Kingdom). The crude extract was centrifuged at 4 °C for 30 min at 40000*g* before loading the soluble fraction on a HisTrap HP 1 mL column (Cytiva, United States), pre-equilibrated in 50 mM sodium phosphate pH 7.0, 10% v/v glycerol. Following extensive washes in the same buffer, 6xHis-*Mm*GMAS was eluted by 50 mM sodium phosphate pH 7.0, 300 mM imidazole, and 10% v/v glycerol (0%–100% linear gradient in 30 column volumes) using a GE AKTA Purifier 100 FPLC System. Fractions containing mostly pure *Mm*GMAS, as revealed by SDS-PAGE analysis (Figure S12), were pooled and buffer-exchanged to 50 mM sodium phosphate, pH 7.0, 10% v/v glycerol using PD-10 desalting columns (Cytiva, United States). The 6xHisTag was removed by thrombin cleavage, incubating 6xHis-*Mm*GMAS overnight with thrombin (Merck Life Sciences, Germany), 20:1 weight ratio. Then, *Mm*GMAS was further purified by Size Exclusion Chromatography (SEC) using a Superdex 200 26/60 column (Cytiva, United States) in the same buffer.

### Differential Scanning Fluorimetry (DSF)

Differential Scanning Fluorimetry (DSF) was performed to evaluate the apparent unfolding temperature of recombinant *Mm*GMAS in different buffers (50 mM Tris pH 8.0, PBS pH 7.0, and Tricine pH 7.5) and in the presence of different additives (15% and 30% v/v glycerol and ethylene glycol; 5% and 10% w/v sucrose). Measurements were performed in duplicate using the exogenous SYPRO orange fluorogenic dye (Thermo Fisher Scientific, United States) and according to the protocol previously described in Fogal et al., 2015.^45^

### Protein Crystallization and X-Ray Data Analysis

Purified *Mm*GMAS was concentrated to 12 mg/mL by a Vivaspin (Sartorius, United Kingdom) ultrafiltration centrifugal device and supplemented with 3 mM MgCl_2_ and 1.4 mM ATPγS, before large protein crystallization screening samples (PACT Premier, Morpheus, JCSG Plus, LMB; Molecular Dimensions Ltd., United Kingdom) were dispensed in the sitting-drop isothermal vapor diffusion setup. Drops containing equal volumes of protein and reservoir solution (0.4 μL total volume) were dispensed on MRC 96-Well 2-Drop Crystallization plates and incubated at 20 °C. The best-diffracting crystals were obtained in the Morpheus A2 precipitant buffer (0.03 M MgCl_2_·6H_2_O, 0.03 M CaCl_2_·2H_2_O, 20% v/v ethylene glycol, 10% w/v PEG 8000, 0.1 M imidazole, 0.1 M MES monohydrate, pH 6.5) and were not further cryoprotected before freezing in liquid nitrogen. X-ray diffraction data (doi: 10.15151/ESRF-ES-1581819979) were collected at the ID30A-3 beamline of the European Synchrotron Radiation Facility (ESRF, Grenoble, France). After several trials, a data set in the *C*121 space group automatically processed by the Xia2/DIALS pipeline was phased by molecular replacement via Molrep using an AlphaFold-generated model of *Mm*GMAS as the starting model.^46–48^

Further data reduction was carried out by Aimless via the CCP4i2 interface.^49,50^ The molecular model was refined automatically by PDB-Redo and REFMAC5 and manually by Coot.^51–53^

Six protein chains (A–F) are present in the asymmetric unit (ASU) and clearly visible in the electron density map from residue Ser1 (chains A–C), Glu2 (chain D), or Met1 (chains E and F) to the C-terminal residue Tyr444. The homo-dodecameric biological assembly of *Mm*GMAS can be reconstructed by applying the crystallographic symmetry operators. While chains A–D of the ASU belong to the same dodecamer, chains E and F belong to an adjacent one.

Protein–protein and protein–ligand interactions were analyzed by PDBePISA (https://www.ebi.ac.uk/pdbe/pisa/) and PLIP (https://plip-tool.biotec.tu-dresden.de/plip-web/plip/index).^54,55^ Surface electrostatic potential was calculated by APBS-PDB2PQR (https://server.poissonboltzmann.org/), simulating an environment with pH 7.0 and 0.15 M NaCl.^56^

### Modeling and Molecular Dynamics Simulations

Two *Mm*GMAS subunits belonging to the same hexameric ring were modeled in complex with 3 Mg^2+^, ADP, methylamine (MMA), and l-γ-glutamyl phosphate (P-γ-GLU) by Boltz-1 via the Tamarind Bio web portal (https://www.tamarind.bio/).^57^ To ensure that a dimer of laterally interacting *Mm*GMAS chains was modeled and to reduce the computational resources for subsequent molecular dynamics, the *Mm*GMAS sequence was trimmed at residue 424.

Conversely, to model MMA in the previously proposed binding site, it was docked on *Mm*GMAS-ADP-P-γ-GLU by AutoDock Vina via the SwissDock webserver.^37,58,59^

Molecular dynamics simulations were performed by Gromacs 2022.3 using the Charmm36-jul2021 force field.^60–62^ Ligands (MMA, ADP, and P-γ-GLU) were parametrized by CGenFF.^63^ Simulations of 800 ns with explicit solvent were performed as previously described.^64,65^ Briefly, the models were solvated in a rectangular box using the TIP3P water model, ensuring a minimum distance of 1 nm between the protein complex and the box boundaries. 0.15 M Na^+^ and Cl^–^ ions were added to neutralize the system net charge and simulate a physiological ionic strength. The system was first minimized with a tolerance of 1000 kJ mol^–1^ nm^–2^ allowing a maximum of 500000 iterations of steepest descent. Subsequently, the system was heated from 0 to 100 K during a 200 ps NVT MD simulation with positional restraints of 400 kJ mol^–1^ nm^–2^. Then, the system was heated to 310 K in 400 ps during an NPT simulation with further lowered restraint (200 kJ mol^–1^ nm^–2^) and further equilibrated over a 5 ns NPT simulation with backbone restraints of 50 kJ mol^–1^ nm^–2^. All restraints were removed for the 800 ns production run. The V-rescale thermostat was used to equilibrate the temperature, whereas the C-rescale barostat was used to control the pressure. Newton’s equation of motion was integrated using a leapfrog algorithm with a 2 fs time step. The particle mesh Ewald (PME) method was used to compute the long-range electrostatic force. Rotational and translational motions of the system were removed, and all bonds were constrained with the LINCS algorithm.

Simulation convergence was assessed by analyzing the global Root Mean Square Deviation (RMSD). Interatomic distances were calculated by using the Gromacs built-in pairdist tool.

## Supplementary Material



## Notes

Strain construction details and biochemical/structural characterization of the recombinant enzyme, plasmid map, assessment of the transgenic strain genome and biotransformation products, effect of l-Thea under nitrogen-depleted conditions, structural analysis details, E-factor calculations, and information on oligonucleotides and growth media used in this study (PDF)
